# De Novo Molecular Design of Caspase-6 Inhibitors by a GRU-Based Recurrent Neural Network Combined with a Transfer Learning Approach

**DOI:** 10.3390/ph14121249

**Published:** 2021-11-30

**Authors:** Shuheng Huang, Hu Mei, Laichun Lu, Minyao Qiu, Xiaoqi Liang, Lei Xu, Zuyin Kuang, Yu Heng, Xianchao Pan

**Affiliations:** 1 Key Laboratory of Biorheological Science and Technology (Ministry of Education), College of Bioengineering, Chongqing University, Chongqing 400044, China; shhuang@cqu.edu.cn (S.H.); 202019021051@cqu.edu.cn (M.Q.); 202019021086@cqu.edu.cn (X.L.); 201919021051@cqu.edu.cn (L.X.); 201819021101@cqu.edu.cn (Z.K.); 201819021102@cqu.edu.cn (Y.H.); 2 Department of Medicinal Chemistry, School of Pharmacy, Southwest Medical University, Luzhou 646000, China

**Keywords:** gated recurrent unit, recurrent neural network, machine learning, transfer learning, caspase-6, inhibitor, molecular design

## Abstract

Due to their potential in the treatment of neurodegenerative diseases, caspase-6 inhibitors have attracted widespread attention. However, the existing caspase-6 inhibitors showed more or less inevitable deficiencies that restrict their clinical development and applications. Therefore, there is an urgent need to develop novel caspase-6 candidate inhibitors. Herein, a gated recurrent unit (GRU)-based recurrent neural network (RNN) combined with transfer learning was used to build a molecular generative model of caspase-6 inhibitors. The results showed that the GRU-based RNN model can accurately learn the SMILES grammars of about 2.4 million chemical molecules including ionic and isomeric compounds and can generate potential caspase-6 inhibitors after transfer learning of the known 433 caspase-6 inhibitors. Based on the novel molecules derived from the molecular generative model, an optimal logistic regression model and Surflex-dock were employed for predicting and ranking the inhibitory activities. According to the prediction results, three potential caspase-6 inhibitors with different scaffolds were selected as the promising candidates for further research. In general, this paper provides an efficient combinational strategy for de novo molecular design of caspase-6 inhibitors.

## 1. Introduction

Caspase is a family of cysteinyl aspartate-specific proteases, which plays a critical role in the cell regulatory networks controlling inflammation and programmed cell death [1]. Up to now, 11 functional caspase subtypes (i.e., caspase 1–10, 14) have been found in human encode proteins, of which caspase-1, -4 and -5 are related to inflammatory response, caspase-14 to keratinocyte differentiation and others to apoptosis. The apoptotic caspases are further divided into two subcategories, namely apoptotic initiator and executioner caspases according to their functions in apoptosis processes. The initiator caspases (caspases-2, -8, -9, and -10) can be recruited and activated by either death receptors or apoptosomes, while the downstream executioner caspases (caspases-3, -6, and -7) are responsible for the actual cell destruction [2,3,4].

**Figure 1:**
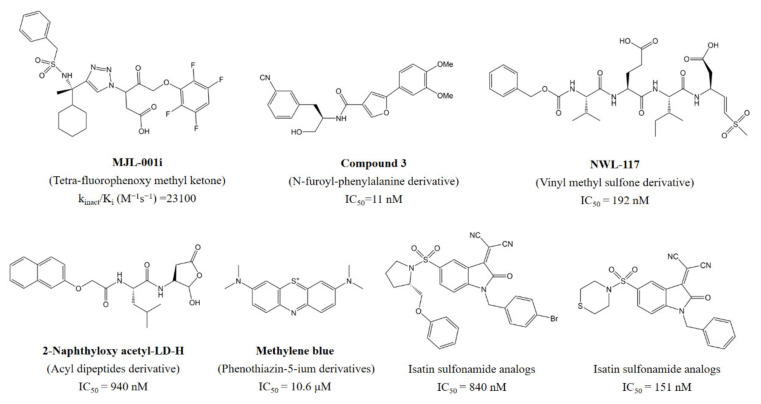
Representative structures of caspase-6 inhibitors.

Accumulated evidence has suggested that the activation of caspase-6 is responsible for neuronal apoptosis and amyloid β peptide (Aβ) deposition, which is highly involved in age-dependent axon degeneration and neurodegenerative diseases, such as Huntington’s disease and Alzheimer’s disease [5,6,7]. Due to the potencies in the treatment of neurodegenerative diseases, caspase-6 inhibitors have attracted intensive attention. Recently, a series of aza-peptides [8], acyl dipeptides [9,10], and non-peptide benzenesulfonyl chloride, isatin sulfonamide [11,12,13,14,15], tetrafluorophenoxy methyl ketone [16], phenothiazin-5-ium derivatives [17], heteroaryl propanamido hexanoic acid [18], vinyl sulfone [19], furoyl-phenylalanine derivatives [20] have been identified as caspase-6 inhibitors with nanomolar to micromolar potencies (Figure 1). However, the existing caspase-6 inhibitors showed more or less inevitable deficiencies that restrict their clinical development and applications. Therefore, there is an urgent need to develop novel caspase-6 candidate inhibitors [21].

Over the last decade, deep learning (DL) technologies, such as convolutional networks (CNN), restricted Boltzmann machines (RBM), recurrent neural networks (RNN), and generative adversarial networks (GAN) have been gradually applied in drug design and proven to be promising approaches for artificial intelligence-based drug design [22,23,24]. Recently, RNN-based molecular generative network has attracted particular attentions duo to its unique features in de novo molecular design [25,26,27]. By using variational auto-encoder (VAE), Gómez-Bombarelli et al. [28] proposed an RNN-based molecular generator which was further applied in a set of drug-like molecules and exhibited excellent predictive power when training jointly with a property prediction task. Winter et al. [29] designed neural network-based translation model and used it to translate chemical structures (e.g., SMILES) into continuous and fixed-sized low-level encodings. Additionally, the models can be used to predict several basic molecular properties for query structures without the need for re-training or including labels.

Olivecrona et al. [30] applied an RNN-based deep learning method combined with policy-based reinforcement learning to generate new molecules with potential activities against dopamine receptor type 2. The results showed that more than 95% of the generated compounds were predicted to be active. Jaques et al. [31] applied RNN and off-policy reinforcement learning methods to generate new molecular structures with desirable properties, such as cLogP and drug-likeness. Although a variety of generative models have been developed for de novo molecular generation, the structural diversity or search space, computational efficiency, and synthetic accessibility, conditional molecule generation, etc. need to be further investigated [32,33].

In this paper, a gated recurrent unit (GRU)-based RNN network combined with transfer learning and traditional machine learning were employed for de novo molecular design of caspase-6 inhibitors. The results showed that the established generative RNN model can generate efficiently potent caspase-6 inhibitors with the similar chemical space distribution to the known caspase-6 inhibitors, which can be easily incorporated with the traditional molecular design methods. In addition, the Surflex-dock method was employed for molecular activities prediction and ranking generated potential inhibitors. Collectively, this paper provides an efficient combinational strategy for de novo molecular design of caspase-6 inhibitors.

## 2. Methods

### 2.1. Datasets

**Figure 2:**
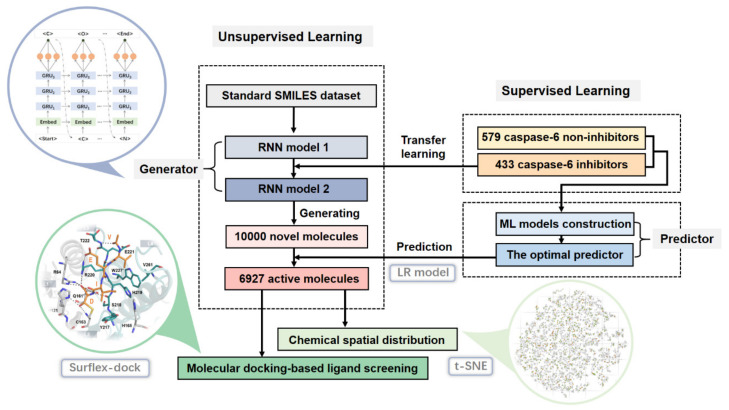
The flowchart of de novo molecular design of the caspase-6 inhibitors.

Figure 2 shows the framework of the de novo design strategy of caspase-6 inhibitors, which mainly consists of 3 parts: (1) the generative RNN network; (2) the ML-based prediction model; (3) molecular docking-based ligand screening. 

In this paper, about 2.4 million chemical molecules including ionic and isomeric compounds were first retrieved from PubChem database [34]. Then, all of the known caspase-6 inhibitors were removed from the dataset. In order to decrease the degree of data heterogeneity, only the molecules with a number of heavy atoms between 10 and 100 and the length of canonical SMILES string less than 140 were selected. As a result, a total of 2,393,029 molecules (SMILES strings) were retained for training the generative RNN network.

To construct a prediction model of caspase-6 inhibitors, 1656 samples consisting of 577 caspase-6 inhibitors and 1079 non-inhibitors were derived from the recent literature (Tables S1 and S2, Supplementary Materials) [9,10,11,12,13,14,15,35,36,37,38,39,40,41,42,43,44,45]. The activities of the collected caspase-6 inhibitors were mainly detected by enzyme inhibition assays and fluorescent plate reader assay.

### 2.2. Machine Learning Based Classification Models of Caspase-6 Inhibitors

Firstly, the 577 caspase-6 inhibitors and 1079 non-inhibitors were divided into a training/validation set (433 positives/579 negatives) and an independent test set (144 positives/500 negatives) according to Table S1. Then, the positive and negative samples in the training/validation set were further randomly divided into the training and validation sets at a ratio of 6:4, respectively. The statistic information of the datasets refers to Table S2. Lastly, a total of 200 fragmental and topological descriptors (Table S3, Supplementary Materials) generated by RDKit toolkit [46] were used for the structural description of the 1656 samples. Herein, five machine learning methods, i.e., support vector machine (SVM), k-nearest neighbor (KNN), Gaussian Naïve Bayesian (GNB), random forest (RF) and logistic regression (LR), were used to construct binary classification models by the Scikit-Learn toolkit [47]. The ROC (receiver operating characteristic), AUC (area under the curve), Matthews correlation coefficient (MCC), accuracy (Acc), specificity (Spe), sensitivity (Sen) and random accuracy (Random Acc) were used for model evaluations [48,49,50].

### 2.3. Generative RNN Modeling and Transfer Learning

**Figure 3:**
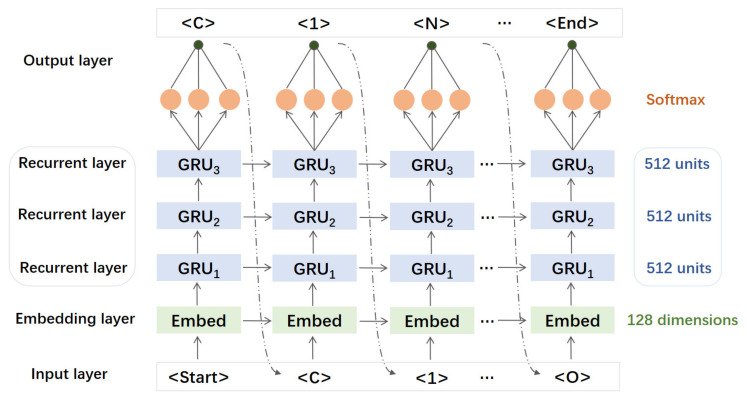
The architecture of the GRU-based recurrent neural network.

The architecture of the generative RNN model is composed of one input layer, one auto-embedding layer with 128 dimensions, three GRU layers with 512 neurons in each layer, and one output layer with softmax activation function (Figure 3). The input layer is responsible for receiving the sequential tokens of the SMILES string of a given sample and the output layer for calculating the occurrence probability of the token at the next position. In this paper, the RNN network was trained by an Adam optimizer [51], of which the initial learning rate is set to 0.001 with a decay rate of 0.05 every 300 steps. The batch size was set to 128 and the loss function was defined as negative log likelihood function. After pretrained by the 2,393,029 SMILES strings from PubChem database, the RNN network was further fine-tuned by using the 433 caspase-6 inhibitors in the training and validation datasets.

### 2.4. Molecular Docking

Surflex-dock (Sybyl 8.1, Tripos Inc., MO, USA) [52] has been proved be an efficient receptor-based drug design and virtual screening strategy, which employs a protomol to guide the generation process of putative ligand binding poses. Herein, a crystal structure of caspase-6 (PDB ID: 3OD5) was used for generating the protomol based on the residues within the 8 Å distance to the co-crystallized ligand Ac-VEID-CHO, a peptidomimetic inhibitor of caspase-6. Before docking, the structures of the ligands were charged by MMFF94 method [53] and then optimized by a Tripos force field [54] with a conjugate gradient minimizer. The maximum iteration steps and energy gradient were set to 10,000 times and 0.05 kcal/mol·Å. To promote the precision of the docking procedure, 3 additional starting conformations per ligand, self-scoring, ring flexibility, soft grid, pre- and post-dock minimizations were also considered in this paper. 

## 3. Results and Discussion

### 3.1. Performances of ML Predictors

**Figure 4:**
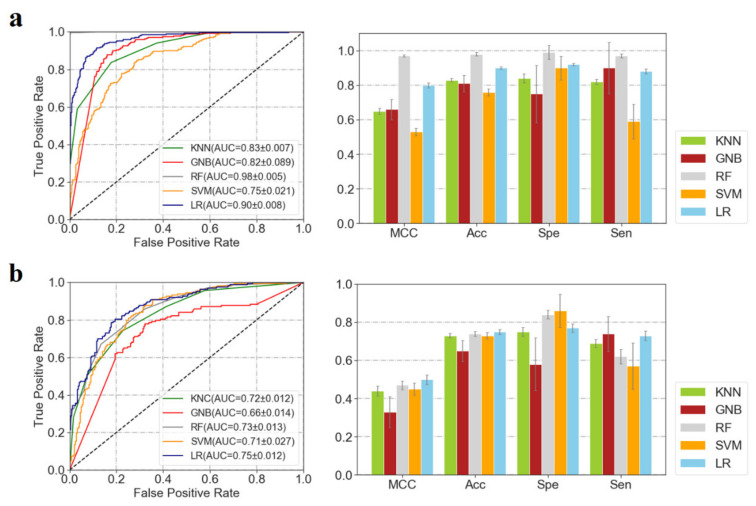
The performances of the 5 ML models on the training (**a**) and validation dataset (**b**). (SVM model: a radial basis function (RBF) kernel was used, of which the C and γ were set as 1 and ‘auto’, respectively; LR model: the inverse of regularization strength, tolerance for stopping criteria, maximum number of iterations, and penalty were set as 0.5, 0.001, 200, and “L1”, respectively. Herein, default parameters were used for the ML models if not specified.)

Herein, ML modeling was performed and repeated 10 times based on the randomly divided training (60%) and validation (40%) sets (Figure 4). It can be observed that most of the ML models showed satisfactory prediction performances on the training and validation datasets. In consideration of the accuracy and balanced performances on the validation set, the LR model was chosen as the optimal predictor, of which the means of AUC, MCC, Acc, Spe and Sen are 0.90 ± 0.008, 0.80 ± 0.015, 0.90 ± 0.008, 0.92 ± 0.007, 0.88 ± 0.014 for the training set, and 0.75 ± 0.012, 0.50 ± 0.025, 0.75 ± 0.013, 0.77 ± 0.023, 0.73 ± 0.025 for the validation set, respectively (Tables S4 and S5, Supplementary Materials). It should be noted that the differences in the prediction performances between the training and validation set may be caused by over-fitting in some degree due to the small training dataset.

Then, five-fold cross-validation and an independent external test by using 644 samples were also performed. The results showed that the optimal LR model achieved excellent prediction performances, of which the Acc for the five-fold cross-validation and the independent test are 0.78 ± 0.047 and 0.86, respectively (Table S6 and Table 1). Therefore, it can be concluded that the resulting LR model is a good predictor of the caspase-6 inhibitors. 

### 3.2. The Generative RNN Modeling

**Figure 5:**
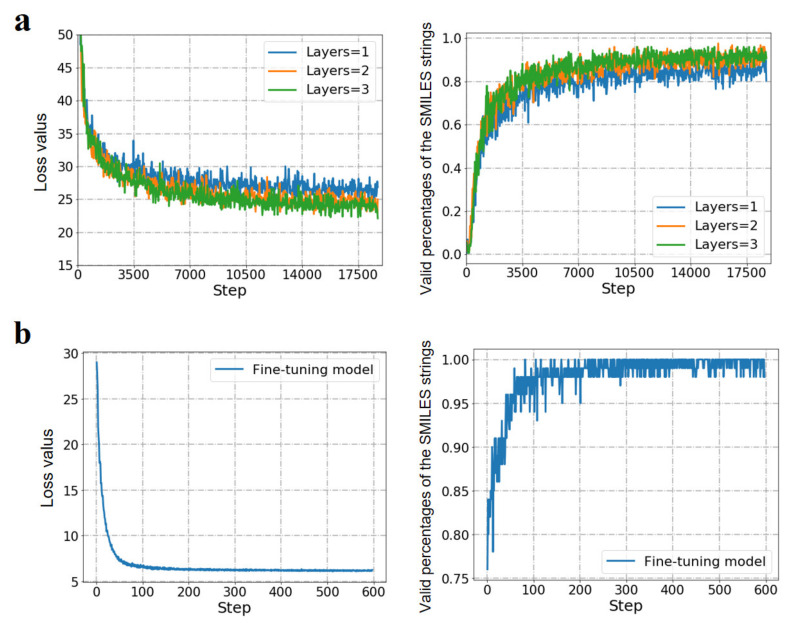
Performances of the pre-trained RNN models with different GRU layers (**a**) and the fine-tuned RNN model by transferred learning of 433 caspase-6 inhibitors (**b**).

Herein, 2,393,029 SMILES strings derived from Pubchem database were used for pre-training of the RNN models. Firstly, the effect of the number of GRU layers on the performance of the generative RNN model was investigated based on the network architecture shown in Figure 3. It can be seen that, after 14,000 steps of iterations, the loss values of the RNN models with one, two and three GRU layers reach the state of convergence (Figure 5a). At the mean time, the valid percentages of 128 SMILES strings sampled by the 3 RNN models reached 0.85, 0.90 and 0.95, respectively. Moreover, no significant improvement in the valid percentage was observed for the RNN models with more than three GRU layers. Thus, the RNN model with three GRU layers was chosen for the following transfer learning. 

In this paper, the 433 caspase-6 inhibitors in the training and validation sets (Table S2) were used for the transfer learning of the pre-trained RNN model. From Figure 5b, it can be observed that, after 200 steps of fine-tuning, the loss value tends to converge and the valid percentage of the sampled SMILES strings reached 99%. In order to evaluate the performance of the refined RNN model in generating potential caspase-6 inhibitors, a retrospective study was performed by using the 144 caspase-6 inhibitors in the test dataset (Table S2), which the RNN model had never seen before. At first, a total of 50,000 valid SMILES strings were randomly sampled by the fine-tuned RNN model. After structural description using the RDKit toolkit, the 50,000 molecules were then predicted by the LR predictor. Based on the predicted positive samples, the recall value of the 144 caspase-6 inhibitors was finally calculated. As shown in Table 2, it can be seen that the percentage of the predicted positive samples remains at a relatively high level during the whole sampling process. Additionally, it can be noticed that the recall value of the 144 caspase-6 inhibitors increases gradually from the lowest value of 2.08% to the highest value of 13.19% (Table 2). Accordingly, it can be concluded that the RNN model can generate efficiently the potential caspase-6 inhibitors after transfer learning. It should be noted that the relatively low recall value is mainly caused by the small sample size of the test caspase-6 inhibitors.

### 3.3. The Distribution in Chemical Space of the Potential Caspase-6 Inhibitors

According to Table 2, a total of 6927 strings (69.3%) were predicted as positive samples from the 10,000 SMILES strings generated. Herein, based on the properties of the H-Bond acceptor/donor, rotatable bonds, aromatic/aliphatic cycles, heterocycle atoms and molecular weight, the distribution of the potential 6927 caspase-6 inhibitors was explored by using the t-distributed stochastic neighbor embedding (t-SNE) method. 

**Figure 6:**
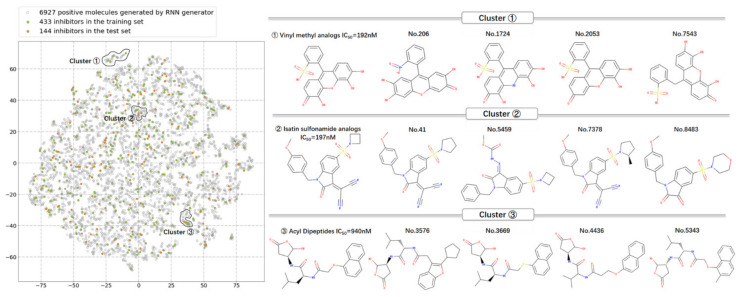
The distribution in the chemical space of the 6927 generated molecules (grey) and 577 known caspase-6 inhibitors (green: training samples; yellow: test samples).

As shown in Figure 6, it can be seen that the distribution of the generated potential 6927 caspase-6 inhibitors in the chemical space is highly overlapped with that of the known 577 caspase-6 inhibitors. Herein, three small clusters of the samples were selected randomly to explore the structural features in detail. For each cluster, it can be observed that the generated molecules have similar molecular scaffolds with the known caspase-6 inhibitors (Figure 6). Thus, it can be inferred that the generated 6927 potential inhibitors have the similar chemical space as the known 577 caspase-6 inhibitors. The structural modification mainly involves substituent modification, scaffold hopping, and chiral transformation, etc., which are also the major means in traditional drug design.

### 3.4. Molecular Docking-Based Ligand Screening

Before docking-based screening of the caspase-6 inhibitors, the protocol of Surflex-dock was first validated by re-docking a co-crystallized ligand Ac-VEID-CHO into the binding pocket of caspase-6 (PDB: 3OD5). The results showed that the Surflex-dock can reproduce the native ligand binding conformation with a docking score of 7.67 (Figure S1, Supplementary Materials).

**Figure 7:**
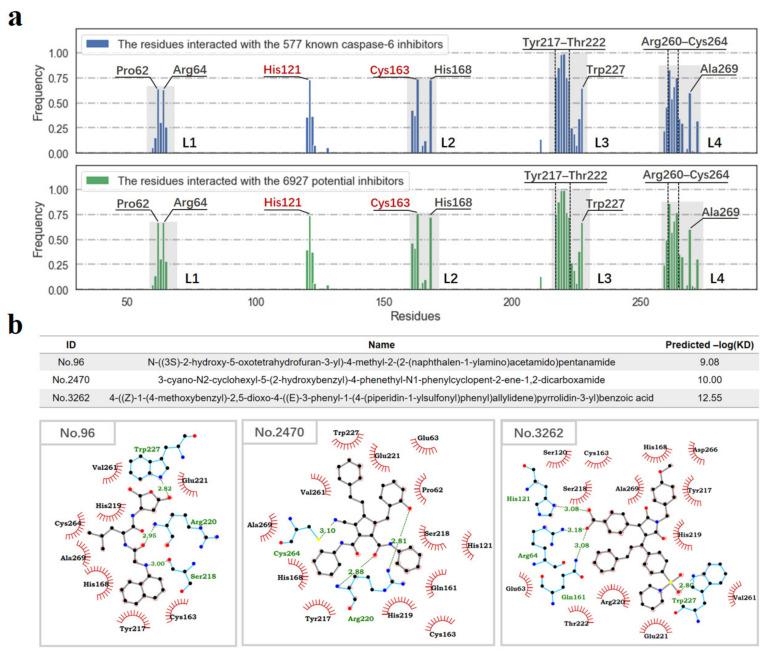
The binding modes of the 577 known caspase-6 inhibitors and 6927 potential inhibitors. (**a**) The occurrence frequencies of the binding residues involved in the intermolecular interactions with the binding ligands (the distance cutoff was set to 5 Å). The residues with the occurrence frequencies larger than 50% are marked, and the catalytic dyad residues His121 and Cys163 are colored in red. (**b**) Schematic diagrams of protein–ligand interactions of three representative samples. H-bonds are represented as green dashed lines. The carbon, nitrogen, oxygen, sulfur atoms are colored in black, blue, red and yellow, respectively.

Based on the docking results of the 577 known caspase-6 inhibitors and the potential 6927 positive samples, the occurrence frequencies of the residues involved in the intermolecular interactions with the 577 caspase-6 inhibitors and 6927 potential inhibitors were investigated, respectively. From Figure 7a, it can be clearly seen that the distributions in the occurrence frequencies of the binding residues are quite similar between the two cases, especially for the binding residues with occurrence frequencies larger than 50%. Therefore, it can be deduced that the potential 6927 inhibitors have similar binding modes with the known 577 caspase-6 inhibitors. 

Furthermore, the Surflex-dock method was employed for predicting and ranking the generated potential inhibitors. Herein, take example for three representative positive samples (ID: 96, 2470 and 3262) with different scaffolds to explore the feasibility of molecular docking-based ligand screening. The docking scores of the 3 positive samples are higher than 9.0 (-logKD), which indicate potential inhibitory activities at nanomolar level. As shown in Figure 7b, both of the sample 96 and 2470 can form strong H-bond interactions with Arg220, while sample 3262 form 3 H-bonds with Arg64, His121 and Gln161. For sample 2470 and 3262, strong π–cation interactions with Arg220 can be also observed. Recent research has proved that Arg64, Gln161, and Arg220 are closely related with the substrate-specificity of caspase-6, and that His121 is a key catalytic residue for substrate hydrolysis [1]. Furthermore, all the three samples can form strong hydrophobic interactions with the hotspot residues Tyr217, Val261, Cys264 and Ala269. Collectively, the three potential caspase-6 inhibitors with nanomolar-level activities are promising candidates for further research.

## 4. Conclusions

In this paper, a GRU-based RNN network combined with transfer learning, ligand-based and receptor-based molecular screening strategies was employed for de novo molecular design of caspase-6 inhibitors. The results showed that the established GRU-based RNN model can accurately learn the SMILES grammars of 2.4 million chemical molecules including ionic and isomeric compounds and is capable of generating novel potential caspase-6 inhibitors with similar chemical space after transfer learning of the known 433 caspase-6 inhibitors. Based on the molecules generated by the RNN models, five ligand-based ML together with the receptor-based docking methods were employed for screening the potential caspase-6 inhibitors. The results showed that the obtained potential caspase-6 inhibitors are mainly generated by substituent modification, scaffold hopping, and chiral transformation, etc. operations from the known inhibitors on the level of SMILES stings. Three potential caspase-6 inhibitors with different scaffolds were finally selected as the most promising candidates for the further research. In general, the framework presented in this paper provides an efficient combinational strategy for de novo molecular design of caspase-6 inhibitors. However, the efficiency and application domain of the proposed molecular design pipeline still need to be tested by in vitro experiments.

## Figures and Tables

**Table 1:** The performance of the optimal LR model on the 644 test samples.

	Confusion Matrix			Performance				
		CP	CN	Acc	Spe	Sen	MCC	Random Acc
Independent test set	PCP	102	49	0.86	0.90	0.71	0.60	0.647
	PCN	42	451					

CP: condition positive; CN: condition negative; PCP: predicted condition positive; PCN: predicted condition negative. For more details, please refer to Table S7 (Supplementary Materials).

**Table 2:** The recall value of the 144 caspase-6 inhibitors.

Sampling Process	I	II	III	IV	V	VI	VII	VIII	IX	X
No. of SMILES strings	1000	2000	3000	4000	5000	10,000	20,000	30,000	40,000	50,000
The predicted positive samples (%)	76.0	72.7	71.4	70.7	70.6	69.3	67.1	66.2	65.5	65.0
Recall (%)	2.08	2.08	3.47	5.55	6.94	8.33	10.41	11.80	13.19	13.19

## Data Availability

Code, data, and pre-trained models are available from our GitHub: https://github.com/ShuhengH/De-Novo-Caspase-6-Inhibitors-Design-by-GRU-Based-RNN-Combined-with-Transfer-Learning-Approach (accessed date: 30 November 2020). Data is contained within the article or Supplementary Material.

## Supplementary Materials

The following are available online at: https://www.mdpi.com/article/10.3390/ph14121249/s1. Table S1: The statistic information of the known caspase-6 inhibitors dataset, Table S2: The information of 1656 samples, Table S3: Definitions of 200 RDKit descriptors, Table S4: The representative confusion matrices of five machine learning models on the training set, Table S5: The representative confusion matrices of five machine learning models on the validation set, Table S6: The 5-fold cross-validation results of the ML models, Table S7: The representative confusion matrices of five machine learning models on test set, Figure S1: The result of molecular docking.
