# Supplementary material for: De Novo Molecular Design of Caspase-6 Inhibitors by a GRU-Based Recurrent Neural Network Combined with a Transfer Learning Approach
Source: Pharmaceuticals (Basel). 2021 Nov 30;14(12):1249. doi: 10.3390/ph14121249 (PMC8706867; doi:10.3390/ph14121249)
Supplement: Supplementary file 1 [file pharmaceuticals-14-01249-s001.zip › Supplementary_Material.pdf]

Supplementary Material

***De Novo* Molecular Design of Caspase-6 Inhibitors by GRU-Based Recurrent Neural Network Combined with Transfer Learning Approach**

Shuheng Huang[1], Hu Mei[1*], Laichun Lu[1*], Minyao Qiu[1], Xiaoqi Liang[1], Lei Xu[1], Zuyin Kuang[1], Yu Heng[1], Xianchao Pan[2*]

[1] Key Laboratory of Biorheological Science and Technology (Ministry of Education), College of Bioengineering, Chongqing University, Chongqing 400044, China

[2] Department of Medicinal Chemistry, School of Pharmacy, Southwest Medical University, Luzhou, Sichuan, 646000, China

Table of contents

	Contents	Page
Table S1	The statistic information of the known caspase-6 inhibitors dataset	S2
Table S2	The information of 1656 samples	S3
Table S3	Definitions of 200 RDKit descriptors	S4-7
Table S4	The representative confusion matrices of five machine learning models on training set	S8
Table S5	The representative confusion matrices of five machine learning models on validationset	S8
Table S6	The 5-fold cross-validation results of the ML models	S9
Table S7	The representative confusion matrices of five machine learning models on test set	S9
Figure S1	Result of molecular docking	S10

Table S1. The statistic information of the known caspase-6 inhibitors dataset

PubChem AID	Training/validation sets		Test set[†]	
	Inhibitors	Non-inhibitors	Inhibitors	Non-inhibitors
49547[1]	-	-	1	-
49549[2]	7	-	2	-
49550[3]	4	-	-	-
49551[4]	-	3	-	-
49555[5]	27	-	10	-
49557[5]	-	-	2	-
49556[6]	12	-	1	-
49558[6]	1	-	-	-
240590[7]	1	-	-	-
241951[8]	1	-	-	-
292077[9]	9	-	4	-
302015[10]	-	-	1	-
412556[11]	-	1	-	-
415378[12]	15	-	2	-
444696[13]	2	-	-	-
591189[14]	18	-	4	-
726063[15]	1	-	-	-
740440[16]	1	-	1	-
1077356[17]	2	-	1	-
1170193[18]	1	-	1	-
652277	-	26	-	-
743332	2	31	-	-
720632	329	518	114	-
686996	-	-	-	500[†]
Total	433	579	144	500

[†] Randomly selected

Table S2. The information of 1656 samples

For more details please refer to the excel file: "TableS2.The_information_of_1656_samples.xlsx"

† Dataset. M: training/validation set; T: test set;

‡ AC_{50}: the concentration causing half-maximal (50%) response;

§ IC_{50}: the concentration causing half-maximal (50%) inhibition.

Table S3. Definitions of 200 RDKit descriptors

No.	RDKit Descriptors	Definition
1	FractionCSP3	The fraction of carbons that are sp3 hybridized
2	NHOHCount	Number of NHs and OHs
3	NOCount	Number of Nitrogen and Oxygen atoms
4	NumAliphaticCarbocycles	Number of aliphatic carbocycles in a molecule
5	NumAliphaticHeterocycles	Number of aliphatic heterocycles in a molecule
6	NumAliphaticRings	Number of aliphatic rings in a molecule
7	NumAromaticCarbocycles	Number of aromatic carbocycles in a molecule
8	NumAromaticHeterocycles	Number of aromatic heterocycles in a molecule
9	NumAromaticRings	Number of aromatic rings in a molecule
10	NumHAcceptors	Number of Hydrogen Bond Acceptors
11	NumHDonors	Number of Hydrogen Bond Donors
12	NumSaturatedCarbocycles	Number of saturated carbocycles in a molecule
13	NumSaturatedHeterocycles	Number of saturated heterocycles in a molecule
14	NumSaturatedRings	Number of saturated rings in a molecule
15	MolLogP	Molecular partition coefficient between aqueous and lipophilic phases [19]
16	MolMR	Molar refractivity of molecule [19]
17	NumHeteroatoms	Number of heteroatoms in a molecule
18	NumRotatableBonds	Number of rotatable bonds in a molecule
19	RingCount	Number of ring in a molecule
20	HeavyAtomCount	Number of heavy atom in a molecule
21	MolWt	The average molecular weight of the molecule
22	HeavyAtomMolWt	Molecular weight of heavy atom
23	NumValenceElectrons	Number of valence electrons
24	NumRadicalElectrons	Number of radical electrons
25	qed	Index for quantitative estimation of drug-likeness
26	ExactMolWt	The exact molecular weight of the molecule
27	MaxPartialCharge	Atomic charges measured by Gasteiger and Marsili [20]
28	MinPartialCharge	
29	MaxAbsPartialCharge	
30	MinAbsPartialCharge	
31	FpDensityMorgan1	Fingerprints density based on the Morgan algorithm, similar to the ECFP/FCFP fingerprints [21]
32	FpDensityMorgan2	
33	FpDensityMorgan3	
34	BalabanJ	Highly discriminating distance-based topological index [22]
35	BertzCT	The general index of molecular complexity [23]

36	HallKierAlpha	Hall-Kier alpha value [24]
37, 38	Chi0, Chi1	Molecular connectivity chi and kappa shape indexes [24]
39-43	Chi0v, Chi1v, Chi2v, Chi3v, Chi4v	
44-46	Kappa1,Kappa2, Kappa3	
47-51	Chi0n, Chi1n, Chi2n, Chi3n, Chi4n	Similar to Hall Kier ChiXv, but uses nVal instead of valence
52	Ipc	The coefficients of the characteristic polynomial of the adjacency matrix of a hydrogen-suppressed graph of a molecule [25]
53	LabuteASA	The approximate molecular van der Waals surface area [26]
54-67	PEOE-VSA1 - PEOE-VSA14	The van der Waals surface area of molecular electrostatic interactions [26]
68-77	SMR-VSA1 - SMR-VSA10	The van der Waals surface area of molecular polarizability [26]
78-89	SlogP-VSA1 - SlogP-VSA12	The van der Waals surface area of molecular hydrophobic and hydrophilic effects [26]
90	TPSA	Molecular topological polar surface area [27]
91-100	VSA-EState1 - VSA-EState10	MOE-type descriptors using electrotopological state indices and surface area contributions (developed at RD, not described in the CCG paper)
101-111	EState-VSA1 - EState-VSA11	
112	MaxEStateIndex	
113	MinEStateIndex	
114	MaxAbsEStateIndex	
115	MinAbsEStateIndex	
116	fr-Al-COO	Number of aliphatic carboxylic acids
117	fr-aldehyde	Number of aldehydes
118	fr-alkyl-carbamate	Number of alkyl carbamates
119	fr-alkyl-halide	Number of alkyl halides
120	fr-allylic-oxid	Number of allylic oxidation sites excluding steroid dienone
121	fr-Al-OH	Number of aliphatic hydroxyl groups
122	fr-Al-OH-noTert	Number of aliphatic hydroxyl groups excluding tert-OH
123	fr-amide	Number of amides
124	fr-amidine	Number of amidine groups
125	fr-aniline	Number of anilines
126	fr-Ar-COO	Number of Aromatic carboxylic acids
127	fr-ArN	Number of N functional groups attached to aromatics
128	fr-Ar-N	Number of aromatic nitrogens
129	fr-Ar-NH	Number of aromatic amines
130	fr-Ar-OH	Number of aromatic hydroxyl groups
131	fr-aryl-methyl	Number of aryl methyl sites for hydroxylation
132	fr-azide	Number of azide groups
133	fr-azo	Number of azo groups

134	fr-barbitur	Number of barbiturate groups
135	fr-benzene	Number of benzene rings
136	fr-benzodiazepine	Number of benzodiazepines with no additional fused rings
137	fr-bicyclic	Number of bicyclic rings
138	fr-C-O	Number of carbonyl
139	fr-C-O-noCOO	Number of carbonyl "O" excluding COOH
140	fr-COO	Number of carboxylic acids
141	fr-COO2	Number of carboxylic acids
142	fr-C-S	Number of thiocarbonyl
143	fr-diazo	Number of diazo groups
144	fr-dihydropyridine	Number of dihydropyridines
145	fr-epoxide	Number of epoxide rings
146	fr-ester	Number of esters
147	fr-ether	Number of ether oxygens (including phenoxy)
148	fr-furan	Number of furan rings
149	fr-guanido	Number of guanidine groups
150	fr-halogen	Number of halogens
151	fr-hdrzine	Number of hydrazine groups
152	fr-hdrzone	Number of hydrazone groups
153	fr-HOCCN	Number of C(OH)CCN-Ctert-alkyl or C(OH)CCNcyclic
154	fr-imidazole	Number of imidazole rings
155	fr-imide	Number of imide groups
156	fr-Imine	Number of Imines
157	fr-isocyan	Number of isocyanates
158	fr-isothiocyan	Number of isothiocyanates
159	fr-ketone	Number of ketones
160	fr-ketone-Topliss	Number of ketones excluding "diaryl" "a,b-unsat"
161	fr-lactam	Number of beta lactams
162	fr-lactone	Number of cyclic esters (lactones)
163	fr-methoxy	Number of methoxy groups 0
164	fr-morpholine	Number of morpholine rings
165	fr-Ndealkylation1	Number of XCCNR groups
166	fr-Ndealkylation2	Number of tert-alicyclic amines
167	fr-NH0	Number of Tertiary amines
168	fr-NH1	Number of Secondary amines
169	fr-NH2	Number of Primary amines
170	fr-Nhpyrrole	Number of H-pyrrole nitrogens

171	fr-nitrile	Number of nitriles
172	fr-nitro	Number of nitro groups
173	fr-nitro-arom	Number of nitro benzene ring substituents
174	fr-nitro-arom-nonortho	Number of non-ortho nitro benzene ring substituents
175	fr-nitroso	Number of nitroso groups excluding NO2
176	fr-N-O	Number of hydroxylamine groups
177	fr-oxazole	Number of oxazole rings
178	fr-oxime	Number of oxime groups
179	fr-para-hydroxylation	Number of para-hydroxylation sites
180	fr-phenol	Number of phenols
181	fr-phenol-noOrthoHbond	Number of phenolic OH
182	fr-phos-acid	Number of phosphoric acid groups
183	fr-phos-ester	Number of phosphoric ester groups
184	fr-piperdine	Number of piperdine rings
185	fr-piperzine	Number of piperzine rings
186	fr-priamide	Number of primary amides
187	fr-prisulfonamd	Number of primary sulfonamides
188	fr-pyridine	Number of pyridine rings
189	fr-quatN	Number of quarternary nitrogens
190	fr-SH	Number of thiol groups
191	fr-sulfide	Number of thioether
192	fr-sulfonamd	Number of sulfonamides
193	fr-sulfone	Number of sulfone groups
194	fr-term-acetylene	Number of terminal acetylenes
195	fr-tetrazole	Number of tetrazole rings
196	fr-thiazole	Number of thiazole rings
197	fr-thiocyan	Number of thiocyanates
198	fr-thiophene	Number of thiophene rings
199	fr-unbrch-alkane	Number of unbranched alkanes of at least 4 members
200	fr-urea	Number of urea groups

Table S4. The representative confusion matrices of five machine learning models on training set

ML models	Confusion matrix			Performance				
		CP	CN	Acc	Spe	Sen	MCC	Random Acc
KNN	PCP	226	61	0.83	0.82	0.84	0.65	0.503
	PCN	43	277					
GNB	PCP	215	44	0.83	0.87	0.80	0.67	0.508
	PCN	54	294					
RF	PCP	258	0	0.98	1.00	0.96	0.97	0.509
	PCN	11	338					
SVM	PCP	159	44	0.74	0.87	0.59	0.49	0.519
	PCN	110	294					
LR	PCP	239	31	0.90	0.91	0.89	0.79	0.506
	PCN	30	307					

*Performances of 5 ML models (Figure 4) were obtained from 10-times repeated ML modeling

CP: condition positive; CN: condition negative; PCP: predicted condition positive; PCN: predicted condition negative.

Table S5. The representative confusion matrices of five machine learning models on validation set

ML models	Confusion matrix			Performance				
		CP	CN	Acc	Spe	Sen	MCC	Random Acc
KNN	PCP	121	55	0.76	0.77	0.74	0.51	0.512
	PCN	43	186					
GNB	PCP	103	51	0.72	0.79	0.63	0.42	0.523
	PCN	61	190					
RF	PCP	107	34	0.77	0.86	0.65	0.50	0.529
	PCN	57	207					
SVM	PCP	97	29	0.76	0.88	0.59	0.50	0.536
	PCN	67	212					
LR	PCP	125	43	0.80	0.82	0.76	0.58	0.516
	PCN	39	198					

CP: condition positive; CN: condition negative; PCP: predicted condition positive; PCN: predicted condition negative.

Table S6. The 5-fold cross-validation results of the ML models

Model	K-fold	ACC	AUC	SPE	SEN
LR	5	0.78±0.047	0.78±0.029	0.80±0.032	0.71±0.034
KNN	5	0.71±0.335	0.77±0.038	0.72±0.034	0.70±0.041
GNB	5	0.58±0.054	0.61±0.064	0.58±0.054	0.58±0.052
RF	5	0.72±0.055	0.75±0.044	0.70±0.026	0.66±0.045
SVM	5	0.66±0.027	0.74±0.060	0.78±0.036	0.63±0.031

*SVM model: a radial basis function (RBF) kernel was used, of which the C and γ were set as 1 and 'auto', respectively; LR model: the inverse of regularization strength, tolerance for stopping criteria, maximum number of iterations, and penalty were set as 0.5, 0.001, 200, and "L1" respectively. Herein, default parameters were used for the ML models if not specified.

Table S7. The representative confusion matrices of five machine learning models on test set

ML models	Confusion matrix			Performance				
		CP	CN	Acc	Spe	Sen	MCC	Random Acc
KNN	PCP	96	72	0.82	0.86	0.67	0.51	0.632
	PCN	48	428					
GNB	PCP	88	102	0.76	0.80	0.61	0.38	0.613
	PCN	56	398					
RF	PCP	86	55	0.82	0.89	0.6	0.49	0.655
	PCN	58	445					
SVM	PCP	82	31	0.86	0.94	0.57	0.56	0.679
	PCN	62	469					
LR	PCP	102	49	0.86	0.90	0.71	0.60	0.647
	PCN	42	451					

CP: condition positive; CN: condition negative; PCP: predicted condition positive; PCN: predicted condition negative.

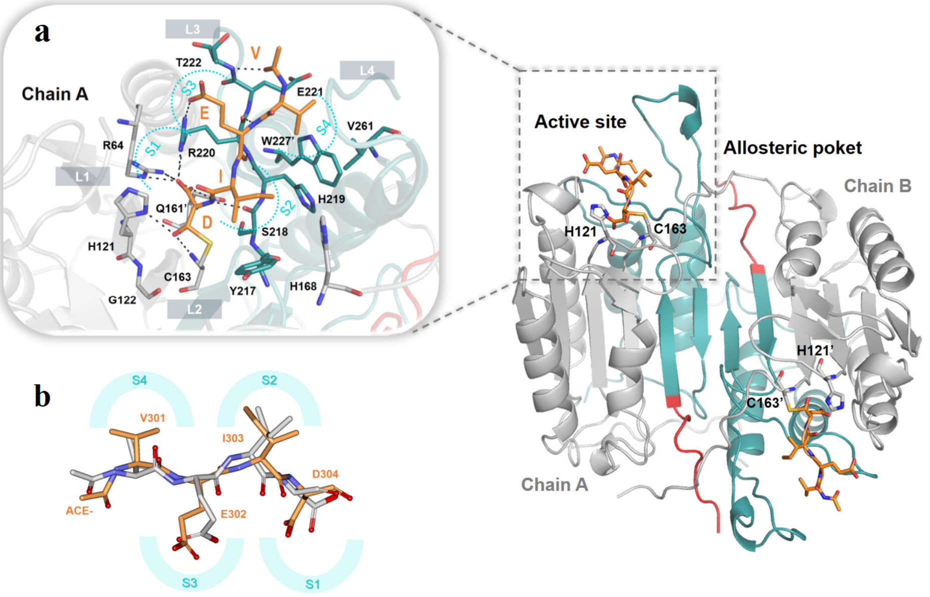

Figure S1. Result of molecular docking. (a) Crucial residues involved in caspase-6 active pocket (PDB ID: 3OD5). The Ac-VEID-CHO and hydrogen bonds are shown in orange and black dashed lines. The large and small subunits are represented in grey and blue, respectively. The parts of the IL domain (loops L2 and L2' residues 198-205) are coloured red. The catalytic dyad residues His121 and Cys163 are represented as sticks. Pocket S1-S4 are shown in blue arcs. (b) Alignments of docking conformation (grey) with naiv̈e conformation (orange). The total score (-log(KD)) and RMSD of the optimal docking conformation were 7.67 and 1.62Å.

References:

1. Wang, Y.; Huang, J. C.; Zhou, Z. L.; Yang, W.; Guastella, J.; Drewe, J.; Cai, S. X., Dipeptidyl aspartyl fluoromethylketones as potent caspase-3 inhibitors: SAR of the P-2 amino acid. *Bioorg. Med. Chem. Lett.* **2004,** *14* (5), 1269-1272.
2. Choong, I. C.; Lew, W.; Lee, D.; Pham, P.; Burdett, M. T.; Lam, J. W.; Wiesmann, C.; Luong, T. N.; Fahr, B.; DeLano, W. L.; McDowell, R. S.; Allen, D. A.; Erlanson, D. A.; Gordon, E. M.; O'Brien, T., Identification of potent and selective small-molecule inhibitors of caspase-3 through the use of extended tethering and structure-based drug design. *J. Med. Chem.* **2002,** *45* (23), 5005-5022.
3. Lee, D.; Long, S. A.; Murray, J. H.; Adams, J. L.; Nuttall, M. E.; Nadeau, D. P.; Kikly, K.; Winkler, J. D.; Sung, C. M.; Ryan, M. D.; Levy, M. A.; Keller, P. M.; DeWolf, W. E., Potent and selective nonpeptide inhibitors of caspases 3 and 7. *J. Med. Chem.* **2001,** *44* (12), 2015-2026.
4. Asgian, J. L.; James, K. E.; Li, Z. Z.; Carter, W.; Barrett, A. J.; Mikolajczyk, J.; Salvesen, G. S.; Powers, J. C., Aza-peptide epoxides: A new class of inhibitors selective for clan CD cysteine proteases. *J. Med. Chem.* **2002,** *45* (23), 4958-4960.
5. Linton, S. D.; Karanewsky, D. S.; Ternansky, R. J.; Wu, J. C.; Pham, B.; Kodandapani, L.; Smidt, R.; Diaz, J. L.; Fritz, L. C.; Tomaselli, K. J., Acyl Dipeptides as reversible caspase inhibitors. Part 1: Initial lead optimization. *Bioorg. Med. Chem. Lett.* **2002,** *12* (20), 2969-2971.
6. Linton, S. D.; Karanewsky, D. S.; Ternansky, R. J.; Chen, N.; Guo, M.; Jahangiri, K. G.; Kalish, V. J.; Meduna, S. P.; Robinson, E. D.; Ullman, B. R.; Wu, J. C.; Pham, B.; Kodandapani, L.; Smidt, R.; Diaz, J. L.; Fritz, L. C.; von Krosigk, U.; Roggo, S.; Schmitz, A.; Tomaselli, K. J., Acyl Dipeptides as reversible caspase inhibitors. Part 2: Further optimization. *Bioorg. Med. Chem. Lett.* **2002,** *12* (20), 2973-2975.
7. Wang, Y.; Guan, L. F.; Jia, S. J.; Tseng, B.; Drewe, J.; Cai, S. X., Dipeptidyl aspartyl fluoromethylketones as potent caspase inhibitors: peptidomimetic replacement of the P-2 alpha-amino acid by a alpha-hydroxy acid. *Bioorg. Med. Chem. Lett.* **2005,** *15* (5), 1379-1383.
8. Han, Y. X.; Giroux, A.; Colucci, J.; Bayly, C. I.; Mckay, D. J.; Roy, S.; Xanthoudakis, S.; Vaillancourt, J.; Rasper, D. M.; Tam, J.; Tawa, P.; Nicholson, D. W.; Zamboni, R. J., Novel pyrazinone mono-amides as potent and reversible caspase-3 inhibitors. *Bioorg. Med. Chem. Lett.* **2005,** *15* (4), 1173-1180.
9. Chu, W. H.; Rothfuss, J.; d'Avignon, A.; Zeng, C. B.; Zhou, D.; Hotchkiss, R. S.; Mach, R. H., Isatin sulfonamide analogs containing a michael addition acceptor: A new class of caspase 3/7 inhibitors. *J. Med. Chem.* **2007,** *50* (15), 3751-3755.
10. Wang, Y.; Jia, S. J.; Tseng, B.; Drewe, J.; Cai, S. X., Dipeptidyl aspartyl fluoromethylketones as potent caspase inhibitors: Peptidomimetic replacement of the P-2 amino acid by 2-aminoaryl acids and other non-natural amino acids. *Bioorg. Med. Chem. Lett.* **2007,** *17* (22), 6178-6182.
11. Thompson, C. M.; Quinn, C. A.; Hergenrother, P. J., Total Synthesis and Cytoprotective Properties of Dykellic Acid. *J. Med. Chem.* **2009,** *52* (1), 117-125.
12. Chu, W. H.; Rothfuss, J.; Chu, Y. X.; Zhou, D.; Mach, R. H., Synthesis and in Vitro Evaluation of Sulfonamide Isatin Michael Acceptors as Small Molecule Inhibitors of Caspase-6. *J. Med. Chem.* **2009,** *52* (8), 2188-2191.
13. Mott, B. T.; Ferreira, R. S.; Simeonov, A.; Jadhav, A.; Ang, K. K. H.; Leister, W.; Shen, M.; Silveira, J.

T.; Doyle, P. S.; Arkin, M. R.; McKerrow, J. H.; Inglese, J.; Austin, C. P.; Thomas, C. J.; Shoichet, B. K.; Maloney, D. J., Identification and Optimization of Inhibitors of Trypanosomal Cysteine Proteases: Cruzain, Rhodesain, and TbCatB. *J. Med. Chem.* **2010,** *53* (1), 52-60.

14. Chu, W. H.; Rothfuss, J.; Zhou, D.; Mach, R. H., Synthesis and evaluation of isatin analogs as caspase-3 inhibitors: Introduction of a hydrophilic group increases potency in a whole cell assay. *Bioorg. Med. Chem. Lett.* **2011,** *21* (8), 2192-2197.

15. Rosse, G., Irreversible Inhibitors of Cysteine Proteases. *ACS Med. Chem. Lett.* **2013,** *4* (2), 163-164.

16. Limpachayaporn, P.; Schafers, M.; Schober, O.; Kopka, K.; Haufe, G., Synthesis of new fluorinated, 2-substituted 5-pyrrolidinylsulfonyl isatin derivatives as caspase-3 and caspase-7 inhibitors: Nonradioactive counterparts of putative PET-compatible apoptosis imaging agents. *Bioorg. Med. Chem.* **2013,** *21* (7), 2025-2036.

17. Krause-Heuer, A. M.; Howell, N. R.; Matesic, L.; Dhand, G.; Young, E. L.; Burgess, L.; Jiang, C. D.; Lengkeek, N. A.; Fookes, C. J. R.; Pham, T. Q.; Sobrio, F.; Greguric, I.; Fraser, B. H., A new class of fluorinated 5-pyrrolidinylsulfonyl isatin caspase inhibitors for PET imaging of apoptosis. *Medchemcomm* **2013,** *4* (2), 347-352.

18. Limpachayaporn, P.; Wagner, S.; Kopka, K.; Schober, O.; Schafers, M.; Haufe, G., Synthesis of 7-Halogenated Isatin Sulfonamides: Nonradioactive Counterparts of Caspase-3/-7 Inhibitor-Based Potential Radiopharmaceuticals for Molecular Imaging of Apoptosis. *J. Med. Chem.* **2014,** *57* (22), 9383-9395.

19. Wildman, S. A.; Crippen, G. M., Prediction of physicochemical parameters by atomic contributions. *J. Chem. Inf. Comput. Sci.* **1999,** *39* (5), 868-873.

20. Gasteiger, J.; Marsili, M., Iterative Partial Equalization of Orbital Electronegativity - a Rapid Access to Atomic Charges. *Tetrahedron* **1980,** *36* (22), 3219-3228.

21. Rogers, D.; Hahn, M., Extended-Connectivity Fingerprints. *J. Chem. Inf. Model.* **2010,** *50* (5), 742-754.

22. Balaban, A. T., Highly Discriminating Distance-Based Topological Index. *Chem. Phys. Lett.* **1982,** *89* (5), 399-404.

23. Bertz, S. H., The first general index of molecular complexity. *J. Am. Chem. Soc.* **1981,** *103* (12), 3599-3601.

24. Hall, L. H.; Kier, L. B., The molecular connectivity chi indexes and kappa shape indexes in structure-property modeling. *Reviews in computational chemistry* **1991,** *2*, 367-422.

25. Bonchev, D.; Trinajstic, N., Information-Theory, Distance Matrix, and Molecular Branching. *J. Chem. Phys.* **1977,** *67* (10), 4517-4533.

26. Labute, P., A widely applicable set of descriptors. *J. Mol. Graph. Model.* **2000,** *18* (4-5), 464-477.

27. Ertl, P.; Rohde, B.; Selzer, P., Fast calculation of molecular polar surface area as a sum of fragment-based contributions and its application to the prediction of drug transport properties. *J. Med. Chem.* **2000,** *43* (20), 3714-3717.
